# Case Report: A novel *EPAS1* mutation in a case of paraganglioma complicated with polycythemia and atrial septal defect

**DOI:** 10.3389/fendo.2023.1180091

**Published:** 2023-07-28

**Authors:** Haiyan Yang, Yue Chen, Kai Liu, Liming Zhao

**Affiliations:** ^1^ Department of Cardiology, West China Hospital, Sichuan University, Chengdu, Sichuan, China; ^2^ Department of Geriatrics, Chongqing General Hospital, Chongqing, China; ^3^ Department of Clinical Medicine, West China Hospital, Sichuan University, Chengdu, Sichuan, China; ^4^ Department of Pharmacy, West China Tianfu Hospital, Sichuan University, Chengdu, Sichuan, China; ^5^ Department of Cardiology, Hospital of Chengdu Office of People’s Government of Tibetan Autonomous Region, Chengdu, Sichuan, China

**Keywords:** paraganglioma, EPAS1, hypertension, atrial septal defect, polycythemia

## Abstract

**Background:**

Paraganglioma is a rare neuroendocrine tumor and is highly associated with hereditary susceptibility genes, often occurring as part of a genetic syndrome. The genetic heterogeneity of paraganglioma poses challenges in diagnosis, counseling, and clinical management.

**Case summary:**

We present the case of a 60-year-old woman with hypertension, atrial septal defect, and polycythemia, who experienced paroxysmal palpitations, sweating, headache, abdominal pain, nausea, and vomiting. Her blood pressure was severely unstable. Blood laboratory tests revealed elevated catecholamine levels, contrast-enhanced CT of her whole abdomen showed a round retroperitoneal mass with soft tissue density, and somatostatin receptor imaging (68Ga PET-CT) indicated a retroperitoneal mass with abnormally increased expression of somatostatin receptor. It is interesting to note that whole exome sequencing (WES) analyses on both blood and tumor samples revealed a novel *EPAS1* mutation, specifically the c.2501A > G; p.Tyr834Cys variant, which has never been reported. The patient was diagnosed with paraganglioma and underwent successful Da Vinci robot-assisted laparoscopic resection of the retroperitoneal tumor. During a 3-month follow-up period, her blood pressure stabilized, and her symptoms significantly improved.

**Conclusion:**

This case reveals that the *EPSA1* mutation may be the primary driver of paraganglioma complicated by atrial septal defect and polycythemia. Additionally, the utilization of Da Vinci robot-assisted laparoscopic surgery contributed to a favorable prognosis for the patient.

## 1 Introduction

Paraganglioma is a rare neuroendocrine tumor arising from chromaffin tissues in the extra-adrenal sympathetic and parasympathetic nervous systems outside the adrenal glands (1, 2). The clinical signs of paraganglioma often lack specificity and occur intermittently, including intermittent palpitations, hypertension, and metabolic abnormalities, leading to diagnostic delays. Catecholamine crisis and catecholamine-induced cardiomyopathy (CICMP) secondary to paraganglioma may be life-threatening. Paraganglioma is strongly associated with hereditary susceptibility genes, and it constitutes a part of a genetic syndrome in approximately one-third to one-half of cases (3). Therefore, prevailing guidelines recommend the consideration of genetic testing for all patients suffering from paraganglioma by accredited laboratories (4, 5). The genetic heterogeneity of paraganglioma poses considerable challenges in genetic diagnosis, counseling, and clinical monitoring of patients with this condition (6). In this article, we present an uncommon and critical case of paraganglioma complicated with polycythemia and atrial septal defect. The patient exhibited symptoms of abnormally fluctuating hypertension and heart failure and subsequently developed secondary catecholamine cardiomyopathy. A novel mutation of *EPAS1*, not previously reported, was detected in her blood and tumor samples. Catecholamine cardiomyopathy was significantly improved after the surgical removal of the paraganglioma.

## 2 Case presentation

A 60-year-old woman presented at our institution with paroxysmal palpitations, sweating, headache, abdominal pain, nausea, and emesis. Approximately a decade prior, she experienced recurrent paroxysmal palpitations and diaphoresis without apparent etiology, which showed limited improvement with symptomatic management. One month ago, her palpitations and diaphoresis intensified, accompanied by headache, abdominal pain, nausea, and vomiting, without chest pain or syncope. Transthoracic echocardiography (TTE) performed at a local hospital revealed an atrial septal defect, prompting her admission to our facility. Throughout her hospitalization, her blood pressure exhibited severe instability, with the highest recorded reading reaching 251/168 mmHg and the lowest recorded reading dropping to 70/43 mmHg. Her medical history included a 2-year diagnosis of hypertension, managed with controlled-release nifedipine and irbesartan hydrochlorothiazide tablets, as well as a 1-year use of Tibetan medicine for diabetes and previous surgical treatment for gallstones. The patient is a non-smoker and does not consume alcohol. Her older brother had a history of atrial septal defect, and her parents passed away due to undisclosed causes.

During the physical examination, the patient exhibited a body temperature of 36°C, a heart rate of 114 beats per minute (bpm), a blood pressure of 163/115 mmHg, a respiratory rate of 20 breaths per minute, and a body mass index of 18.7 kg/m^2^. No evidence of hepato-jugular reflux with jugular venous distension was observed, and there were no signs of moist rales or wheezing in either lung field. A cardiac examination revealed tachycardia with a regular rhythm and no audible cardiac murmurs. The abdomen exhibited softness, and the presence of a mass was scarcely palpable. Furthermore, there was no evidence of edema in the lower extremities.

Blood laboratory tests revealed a notable elevation in catecholamine levels, including epinephrine at 7,241.6 pmol/L (normal range, 0–605.4 pmol/L), norepinephrine at 69,645 pmol/L (normal range, 414–4,435.5 pmol/L), dopamine at 575.3 pmol/L (normal range, 0–195.7 pmol/L), metanephrine at 3.01 nmol/L (normal range, 0–0.5 nmol/L), and normetanephrine exceeding 20.56 nmol/L (normal range, 0–0.9 nmol/L). At the same time, the resin–angiotensin–aldosterone system (RAAS), adrenocorticotropic hormone, cortisol, and dehydroepiandrosterone sulfate exhibited normal values. Urinary measurements demonstrated elevated levels of epinephrine at 123.03 nmol/24 h (normal range, 4.31–61.6 nmol/24 h) and norepinephrine at 2,488.95 nmol/24 h (normal range, 60–352 nmol/24 h), while dopamine remained within normal limits. Additionally, Troponin-T levels were elevated at 245.8 pg/ml (normal range, 0–14 pg/ml), CK-MB at 5.74 ng/ml (normal range, 0–2.88 ng/ml), and N-terminal prob-type natriuretic peptide (NT-proBNP) at 11,411 pg/ml (normal range, 0–450 pg/ml). Hematocrit levels in the blood routine indicated 56.7%, with a hemoglobin level of 195 g/L.

**Figure 1:**
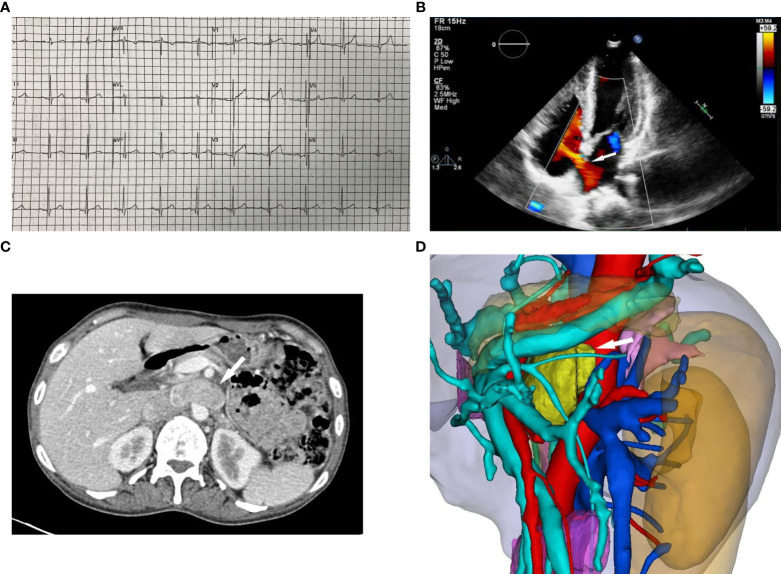
**(A)** 12-lead electrocardiogram shows sinus rhythm, and no significant ST-T changes. **(B)** Transthoracic echocardiography shows the white arrow indicate the atrial septal defect, which shows a discontinuity in the continuity of the middle atrial septum and a shunt from left to right. **(C)** Abdomen contrast-enhanced CT shows the white arrow indicate a round retroperitoneal mass with soft tissue density (about 4.5×2.5cm in size) and marked heterogeneous enhancement. **(D)** 3D reconstruction of the retroperitoneal mass shows the yellow mass indicated by the white arrow is the retroperitoneal mass, the red is the artery, the blue is the vein, and the sky blue is the portal vein. The retroperitoneal mass is closely adjacent to the inferior vena cava, aorta, portal vein, right renal artery and left renal artery.

**Figure 2:**
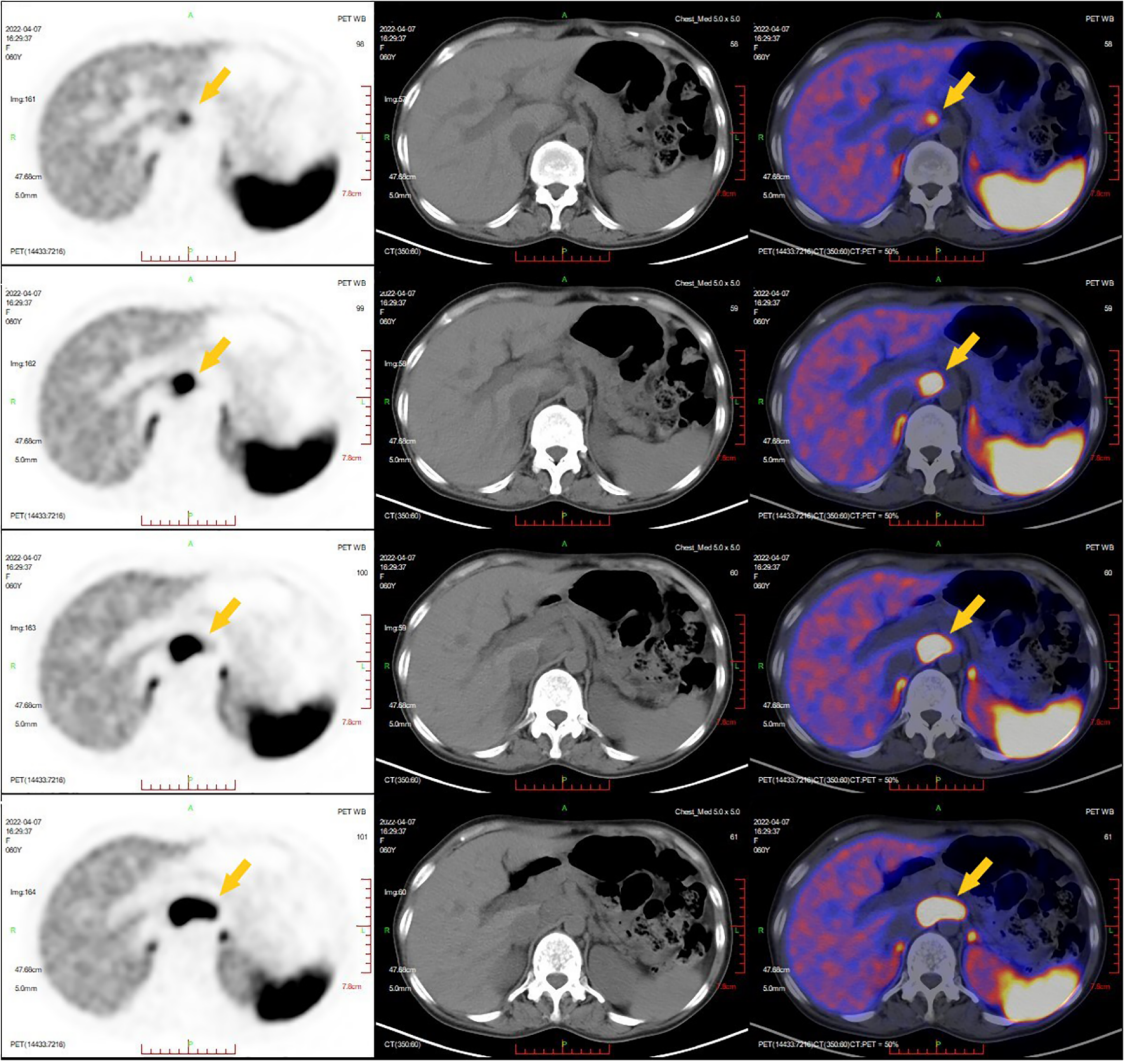
Somatostatin receptor imaging (68Ga PET-CT). The yellow arrow indicated a retroperitoneal mass with abnormal increased expression of somatostatin receptor was consistent with the appearance of paraganglioma.

The 12-lead electrocardiogram demonstrated sinus rhythm (**Figure 1A**). TTE revealed the presence of an atrial septal defect, a thickened ventricular septum measuring 13 mm, mild mitral and tricuspid regurgitation, reduced left ventricular systolic function, and an ejection fraction (EF) of 46% (**Figure 1B**). Contrast-enhanced CT of the entire abdominal region showed a round retroperitoneal mass with soft tissue density, measuring approximately 4.5×2.5 cm, exhibiting notable heterogeneous enhancement. Considering these findings, the possibility of a neurogenic tumor was considered (**Figure 1C**). To better assess the mass’s relationship with adjacent structures, a three-dimensional reconstruction of the retroperitoneal mass in the abdomen was performed, revealing its proximity to the inferior vena cava, aorta, portal vein, right renal artery, and left renal artery (**Figure 1D**). Somatostatin receptor imaging (68Ga PET-CT) indicated abnormally increased expression of somatostatin receptor in the retroperitoneal mass, consistent with the appearance of paraganglioma, and no evidence of tumor metastasis elsewhere in the body (**Figure 2**).

**Figure 3:**
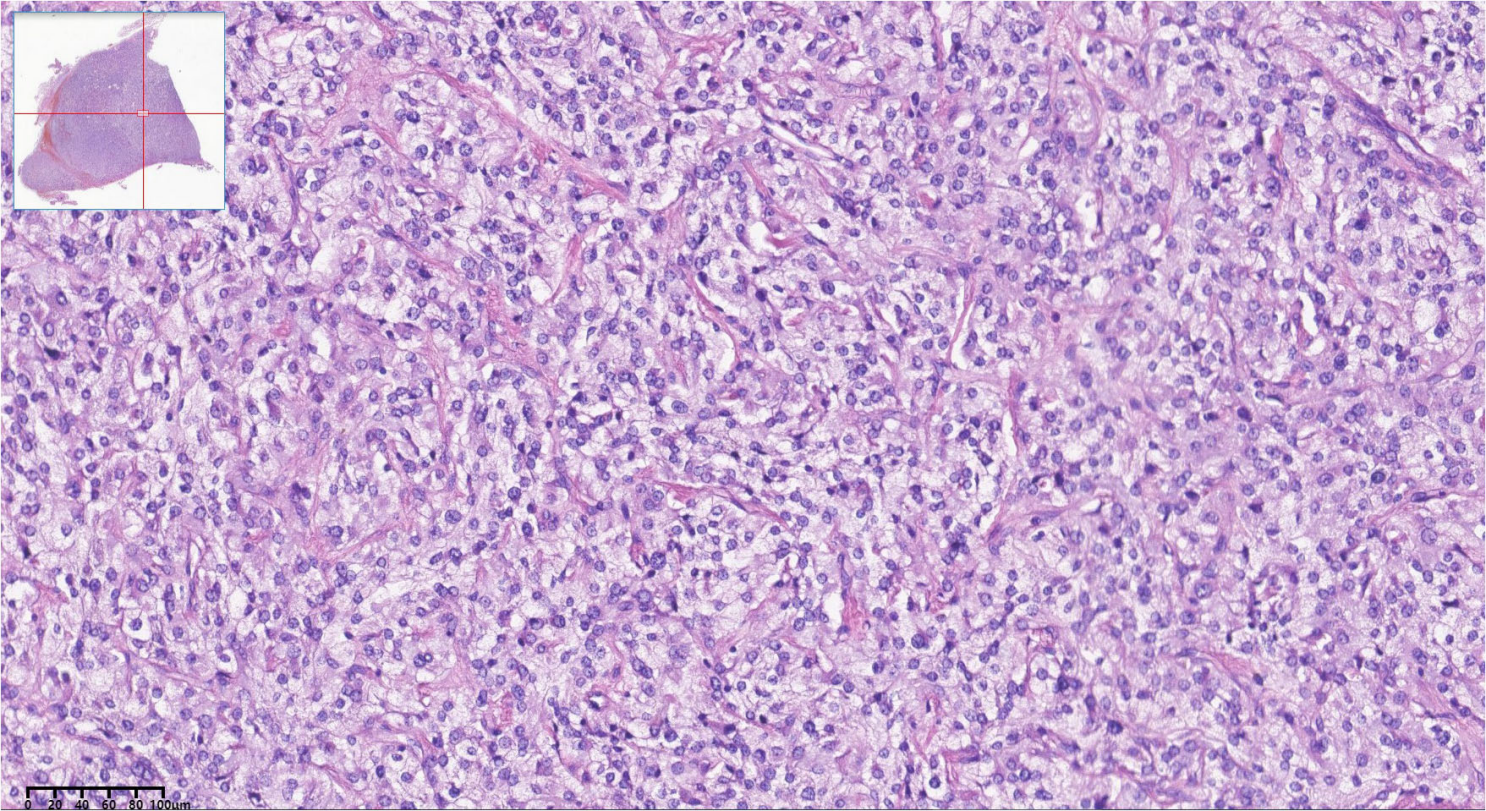
Histopathologic pictures of the retroperitoneal mass. Hematoxylin-eosin staining showed characteristic ‘Zellballen’ architecture of tumor cells.

Upon admission, the patient’s symptoms and examination results led to the diagnosis of paraganglioma, catecholamine cardiomyopathy, hypertensive crisis, atrial septal defect, and diabetes. Treatment was initiated with nifedipine controlled-release tablets, phenoxybenzamine, metoprolol sustained-release tablets, and immediate restoration of circulating volume. Subsequently, the frequency of symptoms such as palpitations, sweating, and headache notably decreased. Additionally, there was a significant improvement in cardiac function, evidenced by a decrease in NT-proBNP from a maximum of 18,128 pg/ml to 187.6 pg/ml, a reduction in Troponin-T from a maximum of 320 pg/ml to 17.6 pg/ml, heart rate fluctuations within the range of 60–70 bpm, and blood pressure maintained below 140/90 mmHg. Following evaluations by cardiologists and urologists, the patient underwent Da Vinci robot-assisted laparoscopic resection of the retroperitoneal tumor. During the operation, the grayish-white tumor measuring 4.0 cm × 3.0 cm was successfully removed with intact encapsulation. Hematoxylin–eosin staining of tumor tissue showed characteristic “Zellballen” architecture of tumor cells. Postoperative pathological examination confirmed the diagnosis of paraganglioma (**Figure 3**).

**Figure 4:**
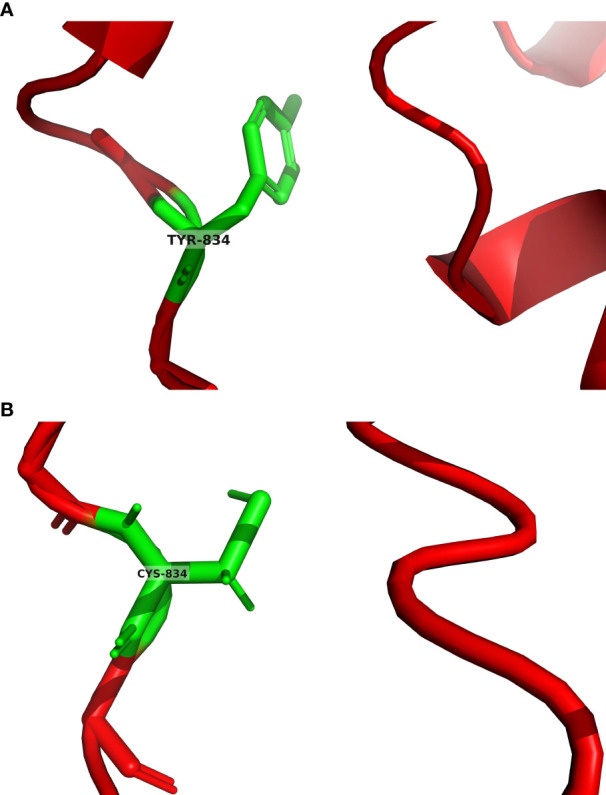
**(A)** EPAS1 wild type was predicted by AlphaFold2. The green color in the figure represents the tyrosine at position 834 of the EPAS1 wild type encoded protein. **(B)** EPAS1 mutant was predicted by tFold. The green color in the figure represents the cysteine at position 834 of the EPAS1 mutant encoded protein.

Owing to the significant association between paragangliomas and hereditary susceptibility genes, whole exome sequencing (WES) was performed on venous whole blood and tumor samples of the patient. All suspicious mutations were identified in both sample types and thus considered as germline ones, and no additional potential somatic mutations associated with pheochromocytomas and paragangliomas were identified with a frequency higher than 5%. This analysis successfully detected germline *EPAS1* mutations, specifically identifying the c.2501A > G; p.Tyr834Cys mutation in the 16th exon of the *EPAS1* gene. Additionally, the patient presented with germline *ACTN2* (c.971G > A; p.Arg3244Gln) and *DSC2* (c.898A > G; p.Thr300Ala) mutations. Concurrently, the patient’s brother provided oral epithelial cells through a buccal swab for WES, revealing consistent gene mutation sites with the patient. We predicted the protein structures of the *EPAS1* wild type and mutant shown on the 3D model (**Figure 4**).

We conducted a 3-month follow-up after the surgery to assess the therapeutic outcomes. Throughout this period, the patient did not experience any episodes of paroxysmal palpitations, diaphoresis, or headaches. The treatment consisting of nifedipine controlled-release tablets, phenoxybenzamine, metoprolol sustained-release tablets, and sacubitril valsartan was continued, resulting in blood pressure control within the range of 125–150/78–95 mmHg, while the heart rate fluctuated between 70 and 85 bpm. Following the resection of paraganglioma, a repeat TTE demonstrated that the interventricular septal thickness had decreased from 13 mm to 12 mm, and the EF had increased from 46% to 63%. These findings indicated a significant improvement in cardiac function following the administered treatment.

## 3 Discussion

Pheochromocytoma and paraganglioma are rare catecholamine-producing neural crest tumors derived from neuroendocrine chromaffin cells. According to a study conducted in Canada, the overall incidence of pheochromocytoma or paraganglioma was 0.66 cases per 100,000 people per year. The prevalence of pheochromocytoma and paraganglioma increased with age, with the highest occurrence observed among individuals aged 60–79 years (7). Most paragangliomas are located in the thoracic and abdominal sympathetic nerves, and retroperitoneal paragangliomas account for more than 50% of all cases, with functional paragangliomas comprising 15%–24% of cases (8). Paragangliomas primarily secrete catecholamines, including epinephrine, norepinephrine, and dopamine. The typical clinical manifestations of paraganglioma are paroxysmal palpitation, headache, sweating, pallor, tremors, and anxiety, often accompanied by episodic or sustained hypertension (9, 10). Neuroendocrine paragangliomas may exhibit atypical symptoms or remain asymptomatic, leading to delayed diagnoses. However, in the case of this patient, the presence of characteristic symptoms such as palpitation, headache, and hypertension, along with significantly elevated catecholamine levels and the identification of a retroperitoneal mass, raised strong suspicion of a functional paraganglioma, facilitating an expeditious confirmation of the diagnosis.

The majority of hereditary paragangliomas are caused by pathogenic variants in genes such as *SDHD*, *SDHB*, *SDHC*, *VHL*, and *NF1* (2). Therefore, patients diagnosed with paraganglioma should undergo *SDH*x mutation testing, while those with metastatic disease should undergo *SDHB* mutation testing. *EPAS1* mutations have been reported in human congenital heart disease and polycythemia (11). Recently, genetic and constitutional mutations of *EPAS1* have been discovered to be connected to the pathogenesis of pheochromocytoma and paraganglioma (12). Gene sequencing of tumor tissue in paraganglioma patients with paraganglioma has revealed that approximately 7% of cases exhibit *EPAS1* mutations, particularly the c.1091A>T (p.Lys364Met) variant. Notably, these mutations were detected in tumor tissues but not in non-neoplastic adrenal tissues. Additionally, mutations of *EPAS1* may contribute to the progression of this group of tumors ascribed to the association of the mutations with high tumor weight and larger tumor size (6). Another study reported a gain-of-function somatic pathogenic variant of *EPAS1* in four of five patients (80%) with pheochromocytoma and paraganglioma who presented with cyanotic congenital heart disease; one of the four patients had paraganglioma with c.1592C>G (p.Pro531Arg) mutation (13). Based on previous studies, the presence of the *EPAS1* mutation in this patient suggests a potential association with paraganglioma, polycythemia, and atrial septal defect. It is encouraging that this variant represents a novel mutation site that has not been reported before.

It is noteworthy that the patient and her brother exhibit two additional genetic variants: *ACTN2* and *DSC2*. These genes are implicated in cardiac abnormalities such as hypertrophic cardiomyopathy, dilated cardiomyopathy, and arrhythmias. Therefore, it is not certain whether they are involved in the mechanism of atrial septal defect formation. However, *ACTN2* and *DSC2* mutations have not been reported to be associated with paraganglioma, atrial septal defect, or polycythemia. This further strengthens our suspicion that the *EPSA1* mutation may be the primary driver of the disease in this patient.

Our case does have certain limitations. The patient’s brother, who also carries the same heterozygous mutation sites and has atrial septal defect without polycythemia, has not exhibited similar paraganglioma symptoms and has not undergone abdominal CT to date. It is unknown whether he possesses a nonfunctional paraganglioma. Consequently, the precise association between the novel *EPAS1* mutation and paraganglioma complicated by polycythemia and atrial septal defect remains inconclusive. Further follow-up is essential to elucidate the clinical significance of this novel mutation site.

Surgical resection of the tumor is the primary treatment for paraganglioma. However, it is crucial to stabilize blood pressure with α adrenoceptor blockers and subsequently introduce β adrenoceptor blockers to stabilize tachycardia before surgery to avoid inducing catecholamine crisis (5). Phenoxybenzamine is often preferred due to its long half-life, compared to phentolamine, which can be started once hemodynamic stability has been achieved. Angiotensin receptor neprilysin inhibitor (ARNI) may also play a role in managing heart failure and hypertension in patients with pheochromocytoma or paraganglioma, as it has been reported to be effective in the follow-up of patients without pheochromocytoma resection (14). Excellent preoperative management strategies combined with optimal surgical methods can minimize surgical complications and improve the prognosis of patients. In this particular case, considering the challenging location of the tumor due to its proximity to the surrounding blood vessels, the urologist choose the Da Vinci robot-assisted laparoscopic surgery. During the procedure, the robotic arm enabled easy access to the tumor. Moreover, the three-dimensional vision clearly delineated the vascular anatomy surrounding the tumor. Compared to standard laparoscopic procedures, Da Vinci robot-assisted laparoscopic surgery offers greater precision, reduced invasiveness, and faster postoperative recovery. It is worth noting that there are limited reports regarding the use of this surgical modality in the treatment of paragangliomas.

## 4 Conclusions

Paraganglioma is a rare neuroendocrine tumor and highly associated with hereditary susceptibility genes. In this case, we have identified a novel *EPAS1* mutation in patients with paraganglioma, which may have significant implications in the pathogenesis of the disease, particularly in cases complicated by atrial septal defect and polycythemia. This finding adds to the growing body of knowledge regarding the genetic basis of paragangliomas. Furthermore, the utilization of Da Vinci robot-assisted laparoscopic surgery has demonstrated its potential to contribute to favorable outcomes in the management of paragangliomas. Although the application of this technique in paraganglioma treatment is relatively limited, it shows promise as an effective surgical modality. Continued follow-up and further research are necessary to better understand the clinical significance of the novel *EPAS1* mutation and its association with paraganglioma, atrial septal defect, and polycythemia.

## Data availability statement

The raw data supporting the conclusions of this article will be made available by the authors, without undue reservation.

## Ethics statement

Written informed consent was obtained from the participant/patient(s) for the publication of this case report.

## Author contributions

HY contributed to writing the original draft. KL contributed to reviewing and editing. LZ and YC contributed to data collection. KL and LZ contributed to conceptualization, resources, and supervision. HY and YC contributed equally to this work and share first authorship. KL and LZ share corresponding authorship. All authors have read and agreed to the published version of the manuscript.

## References

[1] Lenders JW Duh QY Eisenhofer G Gimenez-Roqueplo AP Grebe SK Murad MH. Pheochromocytoma and paraganglioma: an endocrine society clinical practice guideline. J Clin Endocrinol Metab (2014) 99(6):1915–42. doi: 10.1210/jc.2014-1498 24893135

[2] Neumann HPH Young WF Eng C. Pheochromocytoma and paraganglioma. New Engl J Med (2019) 381(6):552–65. doi: 10.1056/NEJMra1806651 31390501

[3] Drovdlic CM Myers EN Peters JA Baysal BE Brackmann DE Slattery WH 3rd. Proportion of heritable paraganglioma cases and associated clinical characteristics. Laryngoscope (2001) 111(10):1822–7. doi: 10.1097/00005537-200110000-00029 11801952

[4] Wachtel H Fishbein L. Genetics of pheochromocytoma and paraganglioma. Curr Opin endocrinol diabetes Obes (2021) 28(3):283–90. doi: 10.1097/MED.0000000000000634 33764930

[5] Garcia-Carbonero R Matute Teresa F Mercader-Cidoncha E Mitjavila-Casanovas M Robledo M Tena I. Multidisciplinary practice guidelines for the diagnosis, genetic counseling and treatment of pheochromocytomas and paragangliomas. Clin Trans Oncol (2021) 23(10):1995–2019. doi: 10.1007/s12094-021-02622-9 PMC8390422 33959901

[6] Islam F Pillai S Gopalan V Lam AK. Identification of novel mutations and expressions of EPAS1 in phaeochromocytomas and paragangliomas. Genes (2020) 11(11):1254. doi: 10.3390/genes11111254 33114456 PMC7693385

[7] Leung AA Pasieka JL Hyrcza MD Pacaud D Dong Y Boyd JM. Epidemiology of pheochromocytoma and paraganglioma: population-based cohort study. Eur J Endocrinol (2021) 184(1):19–28. doi: 10.1530/EJE-20-0628 33112261

[8] Li P Zhao D. A rare case of retroperitoneal paraganglioma-case report and literature review. Trans Gastroenterol Hepatol (2016) 1:58. doi: 10.21037/tgh.2016.06.01 PMC5244753 28138625

[9] Y-Hassan S Falhammar H. Clinical features, complications, and outcomes of exogenous and endogenous catecholamine-triggered Takotsubo syndrome: A systematic review and meta-analysis of 156 published cases. Clin Cardiol (2020) 43(5):459–67. doi: 10.1002/clc.23352 PMC7244299 32125009

[10] Falhammar H Kjellman M Calissendorff J. Initial clinical presentation and spectrum of pheochromocytoma: a study of 94 cases from a single center. Endoc connections (2018) 7(1):186–92. doi: 10.1530/EC-17-0321 PMC5776668 29217652

[11] Pillai S Gopalan V Smith RA Lam AK. Updates on the genetics and the clinical impacts on phaeochromocytoma and paraganglioma in the new era. Crit Rev oncology/hematol (2016) 100:190–208. doi: 10.1016/j.critrevonc.2016.01.022 26839173

[12] Buffet A Burnichon N Favier J Gimenez-Roqueplo AP. An overview of 20 years of genetic studies in pheochromocytoma and paraganglioma. Best Pract Res Clin Endocrinol Metab (2020) 34(2):101416. doi: 10.1016/j.beem.2020.101416 32295730

[13] Vaidya A Flores SK Cheng ZM Nicolas M Deng Y Opotowsky AR. EPAS1 mutations and paragangliomas in cyanotic congenital heart disease. New Engl J Med (2018) 378(13):1259–61. doi: 10.1056/NEJMc1716652 PMC5972530 29601261

[14] Yu M Du B Yao S Ma J Yang P. Von Hippel-Lindau syndrome with a rare complication of dilated cardiomyopathy: a case report. BMC Cardiovasc Disord (2022) 22(1):489. doi: 10.1186/s12872-022-02913-1 36401171 PMC9673439

